# Treatment with intravenous immunoglobulin modulates coagulation- and complement-related pathways in COVID-19 patients

**DOI:** 10.3389/fimmu.2025.1623309

**Published:** 2025-07-31

**Authors:** Dominic McGrosso, Jessica Raygoza, Avnee J. Kumar, Michael T. Y. Lam, Laura A. Barnes, Sophia Karandashova, Alexia Perryman, Matthew Geriak, Mazen F. Odish, Nicole G. Coufal, Brian Lichtenstein, George Sakoulas, Angela Meier, Victor Nizet, Jorge A. Masso-Silva

**Affiliations:** ^1^ Department of Pharmacology, University of California, San Diego, La Jolla, CA, United States; ^2^ Skaggs School of Pharmacy and Pharmaceutical Sciences, University of California, San Diego, La Jolla, CA, United States; ^3^ Pulmonary and Critical Care Section, Veterans Affairs San Diego Healthcare System, La Jolla, CA, United States; ^4^ Department of Medicine, Division of Pulmonary, Critical Care, Sleep and Physiology, University of California, San Diego, La Jolla, CA, United States; ^5^ Division of Respiratory Medicine, Department of Pediatrics, University of California San Diego, La Jolla, CA, United States; ^6^ Sharp Center for Research, San Diego, CA, United States; ^7^ Sanford Consortium for Regenerative Medicine, La Jolla, CA, United States; ^8^ Department of Pediatrics, University of California San Diego, La Jolla, CA, United States; ^9^ Rady Children’s Hospital, San Diego, CA, United States; ^10^ Division of Hospital Medicine, Department of Internal Medicine, Sharp Rees Stealy Medical Group, San Diego, CA, United States; ^11^ Collaborative to Halt Antimicrobial Resistant Microbes (CHARM), Department of Pediatrics, University of California School of Medicine, La Jolla, CA, United States; ^12^ Department of Anesthesiology, Division of Critical Care, University of California, San Diego, La Jolla, CA, United States

**Keywords:** COVID-19, ARDS, intravenous immunoglobulin, IVIg, mass spectrometry, tandem mass tag, coagulation, complement

## Abstract

**Introduction:**

Intravenous immunoglobulin (IVIG) is a therapy that uses pooled immunoglobulins from thousands of different donors. While it is primarily used to treat immunodeficiency and autoimmune diseases due to its immunomodulatory properties, IVIG has also been used as an off-label therapy for respiratory infections, including COVID-19. Clinical data regarding the efficacy of IVIG for COVID-19 has been controversial, and although some smaller studies have shown beneficial effects, others including a large randomized trial found no significant clinical impact but noted detrimental secondary effects.

**Methods:**

We describe the first proteomic analysis from the plasma of COVID-19 patients treated with IVIG, as well as clinical outcomes.

**Results:**

Patients that received IVIG early upon hospitalization have faster clinical improvement. Proteomic analysis showed that serum from patients with COVID-19 has increased levels of proteins associated with inflammatory responses, activation of coagulation and complement pathways, and dysregulation of lipid metabolism. IVIG therapy significantly impacted pathways related to coagulation. Given known crosstalk between coagulation and complement pathways, we also analyzed complement-related proteins. Overall, treatment with IVIG appeared to modulate coagulation (KNG1, ACTB, FGA, F13B, and CPB2) and complement (C1RL, C8G and CFD) related proteins.

**Discussion:**

Our data is supported by similar findings observed in disease states other than COVID-19, where IVIG can impact coagulation and complement proteins. However, early administration seems to be critical determinants to optimize responsiveness to IVIG therapy in COVID-19.

## Introduction

Intravenous immunoglobulin (IVIG) is primarily used in the treatment of autoimmune diseases, including antiphospholipid syndrome, systemic lupus erythematosus, chronic inflammatory demyelinating polyneuropathy, and multiple sclerosis (1, 2). In addition, IVIG may be given prior to transplant to reduce the numbers of allo-antibodies and the risk of antibody-mediated rejection in solid organ transplants (3). IVIG consists of pooled immunoglobulins from several thousand healthy blood donors and is typically infused at high doses (4, 5). Therapeutic responses to IVIG are thought to arise either from antigen neutralization—mediated by the variable region of antibodies binding specific antigens—or from immunomodulation via Fc-mediated interactions with immune cell receptors (6, 7). In this context, IVIG has been applied not only for autoimmune disease indications but also for treating a wide range of infectious and inflammatory conditions. Indeed, off-label use of IVIG for various conditions exceeds its use in those with formal regulatory approval (2, 8–10). Among infectious diseases, IVIG has been used in viral respiratory infections in both immunocompetent and immunocompromised individuals (8, 10).

During the COVID-19 pandemic, IVIG was extensively explored as a potential disease-modulating therapy (8, 11–14) due to its relatively low toxicity profile and broad immune-regulatory effects—without the immunosuppression observed with other treatments such as glucocorticoids, tocilizumab, or baricitinib (15, 16). Nevertheless, the clinical utility of IVIG in COVID-19 has remained controversial, with inconsistent outcomes across studies. Some studies have shown that IVIG, when administered in conjunction with antivirals and glucocorticoids, is associated with shorter hospital stays, fewer days requiring mechanical ventilation or ICU-level care, faster normalization of body temperature, and improved oxygenation (17–24). Positive outcomes have been linked to early administration of IVIG, younger age, and fewer baseline comorbidities (21, 24). A recent study of patients with long COVID-19 suggested that those with marked immune perturbations experienced clinical benefit from IVIG treatment (25). Additional smaller retrospective studies have suggested potential benefit in immunocompromised patients with COVID-19 (26, 27). Conversely, other studies, including a large phase 3 randomized controlled trial found no clinical benefit of IVIG treatment in COVID-19 (28), and some reported potential adverse effects, such as thromboembolism and acute kidney injury (14, 20, 28–34).

IVIG’s mechanism of action is multifactorial and incompletely understood (4). In COVID-19, disease severity is linked to a dysregulated systemic immune response with associated thrombosis, contributing to multi-organ injury and high mortality (35, 36). This includes disruption in complement-activation pathways (37). IVIG has been shown to exert complement scavenging effects, interfering with the deposition and activity of activated complement components (38–41). COVID-19 pathogenesis is also characterized by dysregulated coagulation (42) and “immunothrombosis”, driven by complex interactions between the inflammatory, immune, coagulation, fibrinolytic and complement systems (42, 43). Immunothrombosis involves interplay between circulating immune cells, vascular endothelium, and prothrombotic host factors, both soluble and membrane-bound (43, 44). Prior studies have shown that IVIG can reduce coagulopathies in some autoimmune diseases (42, 45).

Understanding how IVIG modulates the dysregulated complement and coagulation systems in COVID-19 could offer crucial insights and inform future therapeutic strategies. Therefore, in this study, we conducted the first proteomic analysis of plasma from COVID-19 patients treated with IVIG, comparing these profiles to untreated COVID-19 patients and healthy controls to identify key molecular signatures associated with IVIG therapy.

## Methods

### Study subjects

Two groups of patients hospitalized for COVID-19 were assessed: 1) patients treated with IVIG (COVID-19 IVIG; n=18) and 2) untreated controls (COVID-19 controls; n=17). In addition, 13 healthy donors were included, who donated blood once a week for 1–4 weeks after passing daily COVID-19 screening protocols. Inclusion criteria for the COVID-19 patients were confirmed or suspected diagnosis of COVID-19, rapid hypoxemic respiratory failure from acute respiratory distress syndrome (ARDS), and mechanically ventilated status. Patients were excluded from consideration of off-label IVIG for the purposes of this study as well as in the overall off-label allocation of IVIG in our healthcare system during the COVID-19 pandemic if they had pre-existing chronic organ failure, history of stroke, dementia, or active malignancy. Both groups received standard of care diagnostic and therapeutic management contemporary for the period in the pandemic (July 2020-March 2021). No patients in this study were vaccinated for SARS-CoV-2.

A total of 18 COVID-19 IVIG patients were included in the study with intention to treat with off-label IVIG therapy (0.5 g/kg adjusted body weight/day for four days). IVIG was administered ≤72 hours after the onset of mechanical ventilation. After successful therapeutic application of IVIG in five patients, the protocol “IVIG in Patients with Severe COVID-19 Requiring Mechanical Ventilation” (clinicaltrials.gov, NCT04616001) was initiated, wherein the same treatment regimen was accompanied by patient consent for collection and subsequent analysis of blood samples. All untreated COVID-19 patients, or designated family member, and healthy controls provided informed consent in accordance with UCSD Rady Children’s Hospital and VASDHS institutional review board (IRB) approval (190699 and B200003, respectively) and within the Helsinki Declaration of the World Medical Association.

### Clinical parameters

Patient sex, age, body mass index (BMI), pre-existing conditions, time of hospitalization, time of intubation for mechanical ventilation, and length of stay were obtained from the electronic medical record. APACHE II scores were calculated on admission to the ICU and O_2_ saturation (SaO_2_) to fractional oxygenation (FiO_2_) ratios were calculated on days 1, 3, 5, and 8 after the first dose of IVIG.

### Sample collection

Blood samples used in this study were residuals from samples drawn as part of routine bloodwork in the ICU; no additional blood was required for inclusion in this study. Plasma was isolated from whole blood by conventional methods and was stored at -80°C. For IVIG-treated (COVID-19 IVIG) patients, samples were collected a day before treatment (referred to as pre-treatment), on day 3–6 after first IVIG was given (referred to as early treatment), and on day 8-13 (referred to as late treatment). Similarly, samples from COVID-19 controls were taken within the same range of time points based on initiation of mechanical ventilation to match their IVIG-treated counterparts.

### Immunoglobulin depletion and sample preparation for mass spectrometry

Plasma was depleted from HSA/Immunoglobulin using Thermo Scientific™ High Select™ HSA/Immunoglobulin Depletion Mini Spin Columns (cat.#: A36366) prior to proteomics sample prep. 10 μl of plasma was added onto the HAS/lgG depletion column containing a resin slurry. Column was capped, incubated for 10 min at room temperature while vortexed to ensure complete coverage in resin. After incubation, columns were centrifuged in a 2 mL collection tube at 1,000 x *g* for 2 minutes. Collected samples were dried down in a speed-vac for proteomic sample prep. For mass spectrometry analysis, plasma samples were sonicated in buffer containing 6 M Urea, 7% SDS, 50 mM TEAB, and protease inhibitor/PhosStop tablet (Roche), with pH adjusted to 8.1 with phosphoric acid. Samples were reduced, alkylated, and acidified before being mixed per manufacturer’s instructions and loaded on S-Trap columns (Protifi). On-column digestion with trypsin for 3 hours at 47°C followed by elution in 50 mM TEAB, 5% formic acid, and 50%/5% acetonitrile and formic acid. Samples were desalted (Waters, C-18 Seppak), and 50 μg aliquots of each sample dried using a speed-vac. Samples were labeled per manufacturer’s instructions (TMT10plex, Lot VL312003, ThermoFisher).

### LC-LC-MS^n^ proteomics

Basic pH reverse-phase LC, followed by data acquisition through LC–MS^2^/MS^3^, was performed as described (46, 47). Linear gradients of 22% to 35% acetonitrile and 10 mM ammonium bicarbonate were passed on HPLC C18 for 75 minutes columns and the resulting fractions concatenated. Fractions were analyzed using tandem mass spectrometry (MS^3^) on an Orbitrap Fusion MS (ThermoFisher) with an in-line EASY-nLC 1000 (ThermoFisher). Separation and acquisition settings were performed using previously defined methods (48).

### Mass spectrometry data analysis

MS data was screened against the reference proteome for *Homo sapiens* downloaded from Uniprot.com on 1/30/2019 using Proteome Discoverer 2.1, and SEQUEST was used to align MS^2^ spectra against theoretical peptides generated *in silico*. Static modifications included TMT labels on N-termini and lysine residues, and carbamidomethylation of cysteines. Dynamic modifications included oxidation of methionine. A 1% false discovery rate was specified for the decoy database search. Peptide spectral match abundances were first summed to the protein level then normalized against the average for each protein divided by the median of all average protein values. A second normalization step was performed whereby the abundance value for each protein per sample was divided by the median value for each channel which had itself been divided by the overall dataset median. Differentially abundant proteins were identified using π score as determined through a Student’s t-test with or without Welch’s correction. Gene ontology and molecular networks were created using Enrichr (49).

### Statistical analyses

One way ANOVA or Fisher exact tests were used to analyze categorical data, where appropriate. Mann Whitney-U was used to compare medians. T-test were performed in unpaired and paired analyses. In all instances, statistical significance was defined as two-tailed p<0.05, unless otherwise noted.

## Results

### Human subject characteristics

In this study, sex was not considered as a biological variable for the multiple assessments performed. Three cohorts were analyzed: healthy controls, COVID-19 controls (non-IVIG-treated) and COVID-19 IVIG-treated patients. The 13 healthy control subjects included 9 males and 4 females, with a mean age of 41.93 (range 25-70). No significant differences were observed between COVID-19 control and COVID-19 IVIG-treated patients in terms of sex, age, BMI, or APACHEII scores. However, IVIG-treated patients had significantly longer durations of mechanical ventilation and hospital stays (**Table 1**, **Supplementary Figure 1**). Despite this, in-hospital survival was similar between the groups; 84.2% in COVID-19 controls (3 deaths out of 19 patients) and 83.3% for COVID-19 IVIG-treated patients (3 deaths out of 18 patients).

**Table 1:** Human subject characteristics.

Characteristics	COVID-19 controls, n (range)	COVID-19 IVIG-treated, n (range)	p value
Sex			
Female	7	6	0.97
Male	12	12	
Total	19	18	
Age	57.2 (17-88)	52.7 (27-76)	0.43
BMI	31.7 (21.9-48)	35 (22.4-89.8)	0.37
APACHE II Score	15.4 (2-34)	14.3 (8-23)	0.63
Hospital length of stay	17.85 (7-40)	30.44 (17-53)	0.0006
Hospital Days Ventilated	11.3 (3-31)	19.3 (4-44)	0.03
Pre-existing conditions			
Morbid obesity	1	4	
Diabetes 2	4	5	
Hypertension	11	4	
Cancer	0	1	
Asthma	1	2	

### Clinical data and IVIG response

Prior studies suggest that IVIG’s benefit in COVID-19 depends on early administration. We therefore examined clinical outcomes based on the timing of IVIG initiation. Eighteen patients were treated with IVIG (5 before protocol initiation and 13 as part of the clinical trial). IVIG was typically administered within 3–5 days after intubation (mean: 1.9 days post-intubation, range 0–5 days). Median time from admission to intubation for mechanical ventilation was 4.5 days, with a range of 1–19 days. All but one patient received IVIG within 72 hours of mechanical ventilation. Two pre-protocol patients received 3 doses (total 1.5 g/kg), while all others received 4 doses (total 2g/kg). The Type 2 diabetes mellitus was the most common comorbidity (28%) followed by hypertension (22%), morbid obesity (50%, defined as BMI >40 kg/m^2^) (**Table 1**).

**Figure 1:**
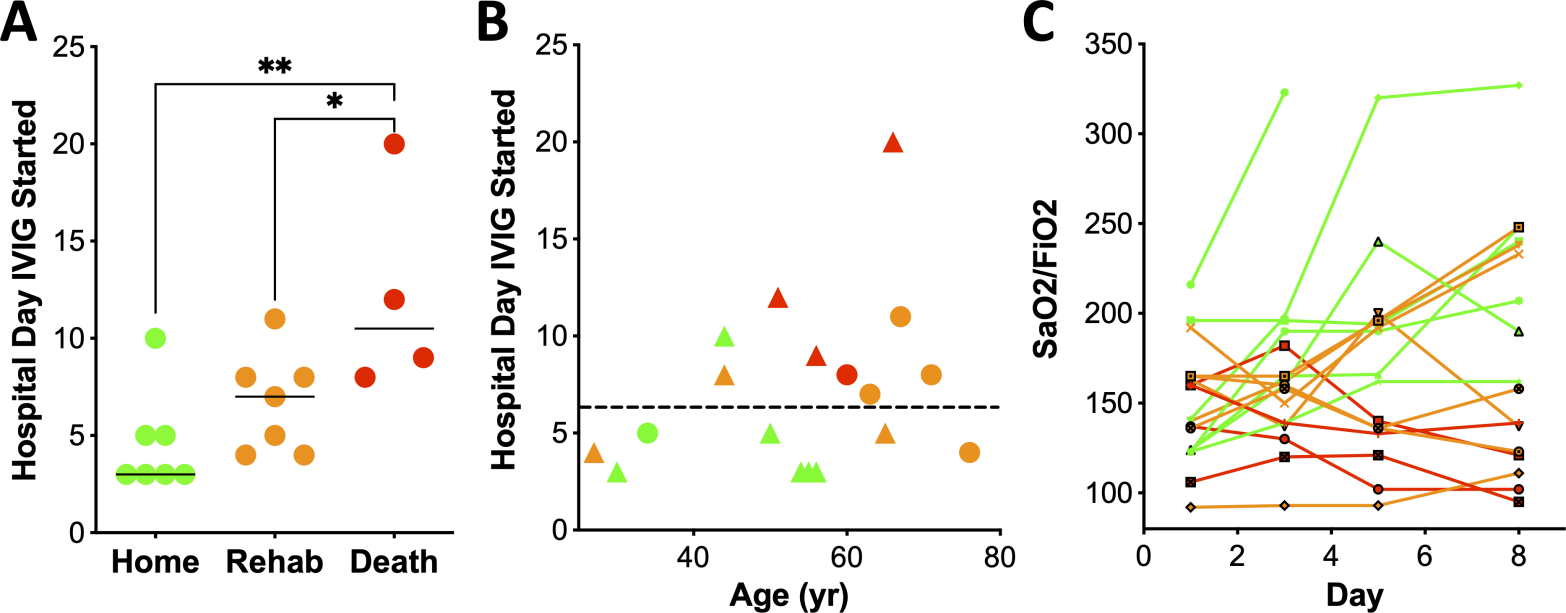
Early treatment with IVIG is associated with improved clinical outcome. **(A)** Day of hospital stay that IVIG treatment was initiated, relative to outcome (one-way ANOVA, Kruskal-Wallis test, p=0.0175). **(B)** Treatment with IVIG within 5 days of hospitalization was associated with discharge to home (p=0.02). Green, orange and red refers to patients sent home, rehab or death, respectively. Circles denote females and triangles males. **(C)** SaO2/FiO2 over time after initiation of IVIG therapy, with day 1 corresponding to the day of the first dose of IVIG. Disposition is noted by color: red (death; n=4), orange (rehabilitation; n=7), and green (home n=7). * p < 0.05, ** p < 0.01.

Earlier IVIG administration was associated with improved clinical outcomes as evidenced by improved functional status at the time of hospital discharge (p=0.0175, **Figure 1A**). Patients discharged home received IVIG at a median of 3 days after hospital admission (range 3-10), with a median hospital stay of 21 days. Those discharged to a rehabilitation received IVIG at a median of 7 days (range 4–11 days), with a median hospital stay of 34 days. For the 4 patients who died, IVIG was administered at a median of 10 days after admission (range 8–20 days). All patients discharged to rehabilitation survived at one year follow-up. There was no difference in age between survivors and non-survivors. Notably, among the patients discharged to a rehabilitation facility were two individuals aged ≥65-years and a 27-year-old with a BMI 90 kg/m^2^ (**Figure 1B**). All patients who initiated IVIG therapy within ≤5 days admission survived, and 67% were discharged home. In contrast, those who received IVIG >5 days after admission, only 11% were discharged home, 44% required rehabilitation, and 44% died. This time-dependent association with mortality reached statistical significance (p=0.041, Fisher exact test).

Lung function also correlated with outcome. Five patients showed rapid improvement in oxygenation after IVIG, defined as stable O_2_ saturation and ≥15% reduction in FiO_2_ within 48 hours of the first dose; all were discharged home (**Figure 1C**). These patients received IVIG earlier (median 3 days, range 3-5) compared to those without improvement (median 8 days, range 3-20, p=0.0146, Mann Whitney-U test, **Figure 1B**). Moreover, patients with improved SaO_2_/FiO_2_ ratios by day 8 all survived, while 4 of the 5 with worsened ratios died. (**Figure 1C**, p=0.002, two-tailed Fisher exact test). One patient was extubated by day 4.

### IVIG treatment modulates plasma proteome

**Figure 2:**
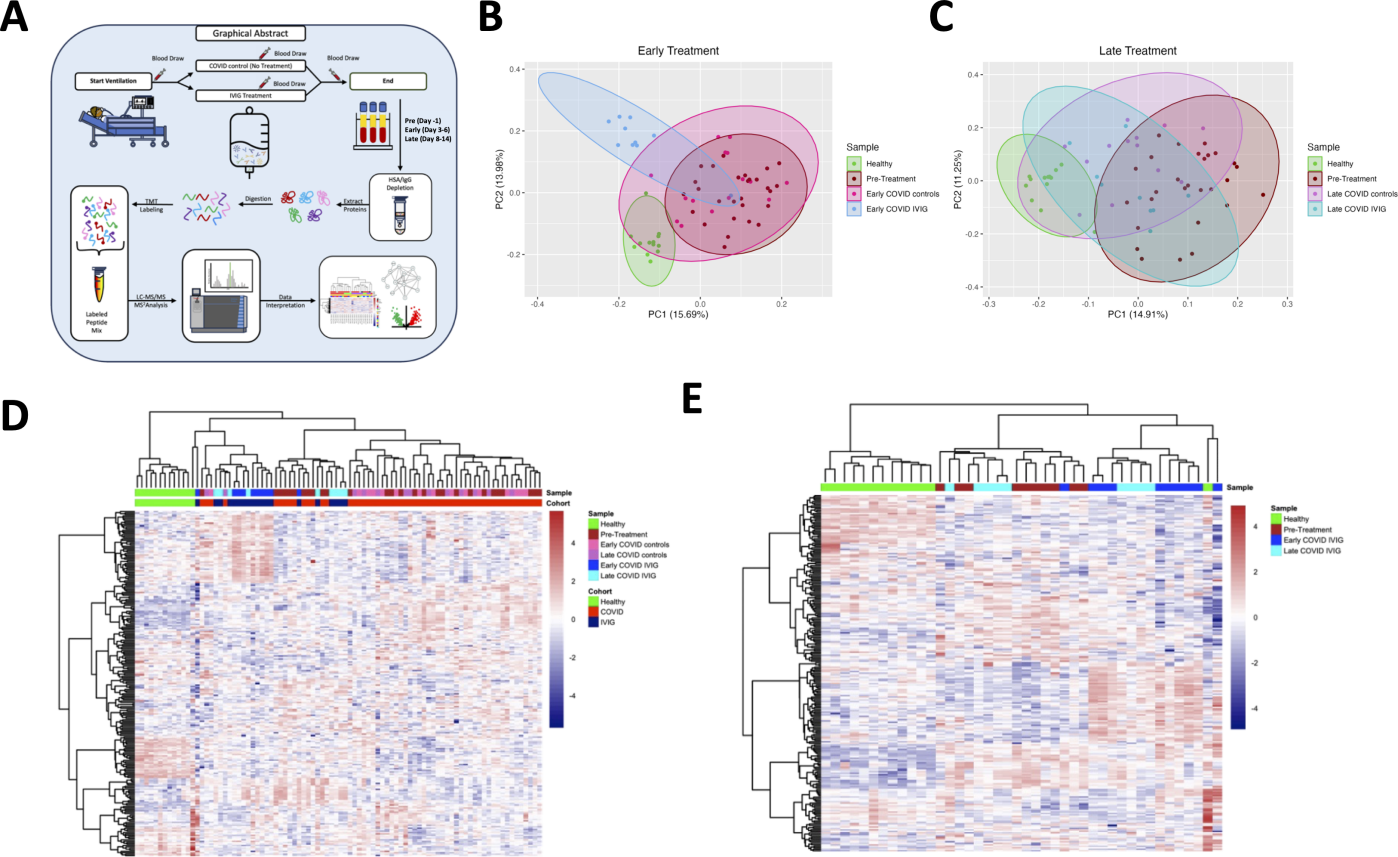
Treatment with IVIG is associated with changes in blood proteome. **(A)** Schematic representation showing the study design. Blood was collected the day before IVIG was started (pre-treatment), at day 3-6 (early) and at day 8-14 (late). Blood samples underwent HAS/IgG depletion, digestion, labeling, mass spectrometry analysis and data interpretation. Principal component analysis of early **(B)** and late samples **(C)** were performed with comparison to pre-treatment. Heatmaps of proteins from COVID-19 controls and subjects treated with IVIG at early and late time points **(D)** and from only IVIG-treated patients **(E)** were created. Both heatmaps include the proteins from pre-treatment and healthy controls.

To determine how IVIG treatment influences the circulating proteome, we performed proteomic analysis of plasma from IVIG-treated COVID-19 patients at multiple time points, as well as from COVID-19 controls and healthy donors. First, we confirmed that disease severity was comparable between COVID-19 control and IVIG-treated groups based on similar APACHE II scores at admission (**Supplementary Figure 1**). **Figure 2A** illustrates the workflow for plasma sample acquisition during hospitalization at early and late time points, including processing steps such as IgG depletion, which was necessary to reduce the dominance of exogenous IVIG in the plasma and allow for detection of other proteins. In addition, **Figure 2A** also outlines the full proteomic pipeline.

We then performed a principal component analysis (PCA) to visualize group differences. As expected, healthy controls clustered distinctly from all COVID-19-infected subjects (**Figure 2B**). Notably, the proteomes of patients receiving early IVIG treatment were different from those of both pre-treatment samples and early COVID-19 controls, which clustered closely together. By contrast, at later timepoints, we observed convergence in the proteomic profiles of all patient groups, with both IVIG-treated and untreated COVID-19 subjects trending closer to healthy controls (**Figure 2C**), consistent with the general clinical improvement seen by that stage.

When we restricted the PCA to only IVIG-treated patients, comparing early versus late treatment time points, we observed a clear temporal evolution in the proteomic profiles: patients sampled at later time points tend clustered closer to healthy controls than those sampled earlier in hospitalization (**Supplementary Figure 2**). These data suggest that IVIG treatment modulates the plasma proteome early after administration, resulting in a profile that differentiates treated patients from untreated COVID-19 controls. However, it is important to highlight that IVIG was only administered for only 4 days, which may explain why some proteomic changes were not sustained at later time points.

Heatmap analyses further highlighted these trends. When comparing proteomic profiles across healthy donors, COVID-19 controls, and IVIG-treated patients, we observed considerable heterogeneity within both COVID-19 groups. Nonetheless, IVIG-treated subjects exhibited proteomic signatures that differed from both healthy donors and COVID-19 controls (**Figure 2D**). This was further supported by a heatmap analysis comparing early versus late samples from IVIG-treated patients, which showed dynamic evolution over time, although some subjects exhibited similar proteomic signatures at both time points (**Figure 2E**). This suggests that while IVIG treatment modulates the plasma proteome in many patients, the response is variable and not uniform across all individuals.

### Treatment with IVIG modulates coagulation and complement pathways

Next, we evaluated specific pathways impacted by IVIG treatment, focusing on differences relative to untreated COVID-19 controls. As a reference point, we first assessed how COVID-19 affected the plasma proteome by comparing early samples from COVID-19 controls (collected day 5 after intubation) with those from healthy donors.

Out of 239 identified proteins, 58 were downregulated and 51 upregulated in COVID-19 controls compared to healthy donors (**Supplementary Figure 3A**). Pathway analysis of these proteins revealed upregulation of inflammatory responses, coagulation and complement activation pathways, as well as disruptions in lipid metabolism (**Supplementary Figure 3B**)—consistent with previous reports of COVID-19 pathophysiology (35, 37, 42, 44, 50, 51). Network analysis highlighted how certain proteins participate in multiple pathways central to COVID-19 pathogenesis, including immune signaling, complement, and coagulation (**Table 2**, **Supplementary Figure 3C**).

**Table 2:** List of proteins associated with coagulation and complement pathways modulated by treatment with IVIG.

Abbreviation	Protein name	Function associated to coagulation or complement	Promote	Inhibit
TNL1	Talin-1	Activates integrin αIIbβ3, enhance platelet adhesion, aggregation, and clot formation, while also regulating cytoskeletal dynamics for stable thrombus formation.	X	
FLNA	Filamin-A	Stabilizes platelet activation, supports integrin-mediated adhesion, and regulates cytoskeletal rearrangements, crucial for thrombus formation and clot stability	X	
FBLN1	Fibulin-1	Stabilizes the extracellular matrix, supports platelet adhesion, and enhances thrombus formation, contributing to clot stability during hemostasis.	X	
ACTB	β-actin	Supports platelet shape changes, adhesion, and aggregation through cytoskeletal rearrangement, essential for thrombus formation and clot stability	X	
VCL	Vinculin (F-actin)	Links integrins to the actin cytoskeleton, facilitating platelet adhesion, spreading, and aggregation, crucial for thrombus formation and clot stability	X	
FGA	Fibrinogen α chain	Facilitates fibrin formation, supports platelet aggregation, and enhance clot stability during hemostasis	X	
FGB	Fibrinogen β chain	Contributes to fibrin polymerization, supports platelet aggregation, and stabilizes clot structure during hemostasis	X	
FGG	Fibrinogen γ chain	Facilitates fibrin clot formation, supports platelet binding, and stabilizes thrombus structure during hemostasis.	X	
VWF	Von Willebrand factor	Mediates platelet adhesion to damaged blood vessels, stabilizes factor VIII, and initiates thrombus formation at injury sites	X	
F2	Coagulation factor II (prothrombin)	Activated as thrombin will activates Factors I, V, VII, VIII, XI, XIII, Protein C and platelets, and thus, it amplifies the coagulation cascade	X	
F5	Coagulation factor V (Proacclerin)	Acts as cofactor of Factor IX-tenase complex, which activates factor X, enhancing thrombin generation and accelerating fibrin clot formation during hemostasis	X	
F9	Coagulation factor IX (Christmas factor, Antihemophilic factor b)	Factor IX-tenase complex activates factor X in the intrinsic pathway, leading to thrombin generation and fibrin clot formation during hemostasis	X	
F13B	Coagulation factor XIII β subunit	Stabilizes fibrin clots through crosslinking fibrin and other matrix proteins, enhancing clot strength and stability	X	
CBP2	Carboxypeptidase B2 (Thrombin-activatable fibrinolysis inhibitor)	Regulates plasminogen activation and clot stability. Inhibits fibrinolysis, supporting clot persistence and inhibits premature breakdown of fibrin	X	
KRT1	Keratin 1	Supports platelet adhesion and clot formation, contributing to thrombus stability and wound healing.	X	
KNG1	Kininogen-1 (HMWK-kallikrein factor, Fitzgerald factor)	Regulates the kallikrein-kinin system, contributes to both coagulation and inflammatory responses during injury; causes increased vascular permeability	X	
THBS1	Thrombospondin-1	Enhances platelet aggregation and stabilizes thrombus formation, while also modulating fibrinolysis and vascular remodeling	X	
HBB	Hemoglobin subunit β	Enhances platelet activation, tissue factor activity, and endothelial dysfunction while impairing fibrinolysis, contributing to thrombosis in hemolytic conditions	X	
APOH	Apolipoprotein H (β2-Glycoprotein1)	Promotes platelet activation and modulates the coagulation cascade, while exhibiting anticoagulant properties by inhibiting excessive thrombin generation	X	X
PRDX2	Peroxiredoxin 2	Reduces oxidative stress, limiting platelet activation, modulating fibrin clot stability, and protecting endothelial function, thereby preventing excessive thrombosis		X
SERPINA1	Serine protease inhibitor A1 (α-1 antitrypsin)	Provides the majority of the antiproteinase activity in human serum, inhibits factor II, factor Xa, active protein C, plasmin, and urokinase		X
SERPINC1	Serine protease inhibitor C1 (antithrombin III)	Binds to and inactivates thrombin and other serine proteases, such as inhibits factor II, factor IXa, and factor Xa, limiting clot formation		X
PROC	Vitamin K-dependent protein C	Degrades factors Va and VIIIa, reducing thrombin generation and promoting an anticoagulant response when activated by thrombin-thrombomodulin		X
C1RL	Complement C1r-like protein	Thought to promote complement-related pathways by contributing to proteolytic processes, but its exact role in complement activation or regulation remains unclear	X	
CFD	Factor D	Cleaves factor B in the alternative pathway, leading to the formation of the C3 convertase and amplifying complement-mediated immune responses	X	
C8G	Complement C8 γ Chain	Stabilizes the C8 complex, facilitating membrane attack complex (MAC) formation, and enhancing target cell lysis	X	
CFHR5	Complement factor H-related protein 5	Competes with factor H, reducing complement regulation, and enhancing C3 convertase activity, leading to increased complement deposition	X	
CD5L	CD5 antigen-like	Regulates the function of C1q, reduces excessive complement activation and prevents inflammation-driven tissue damage		X

**Figure 3:**
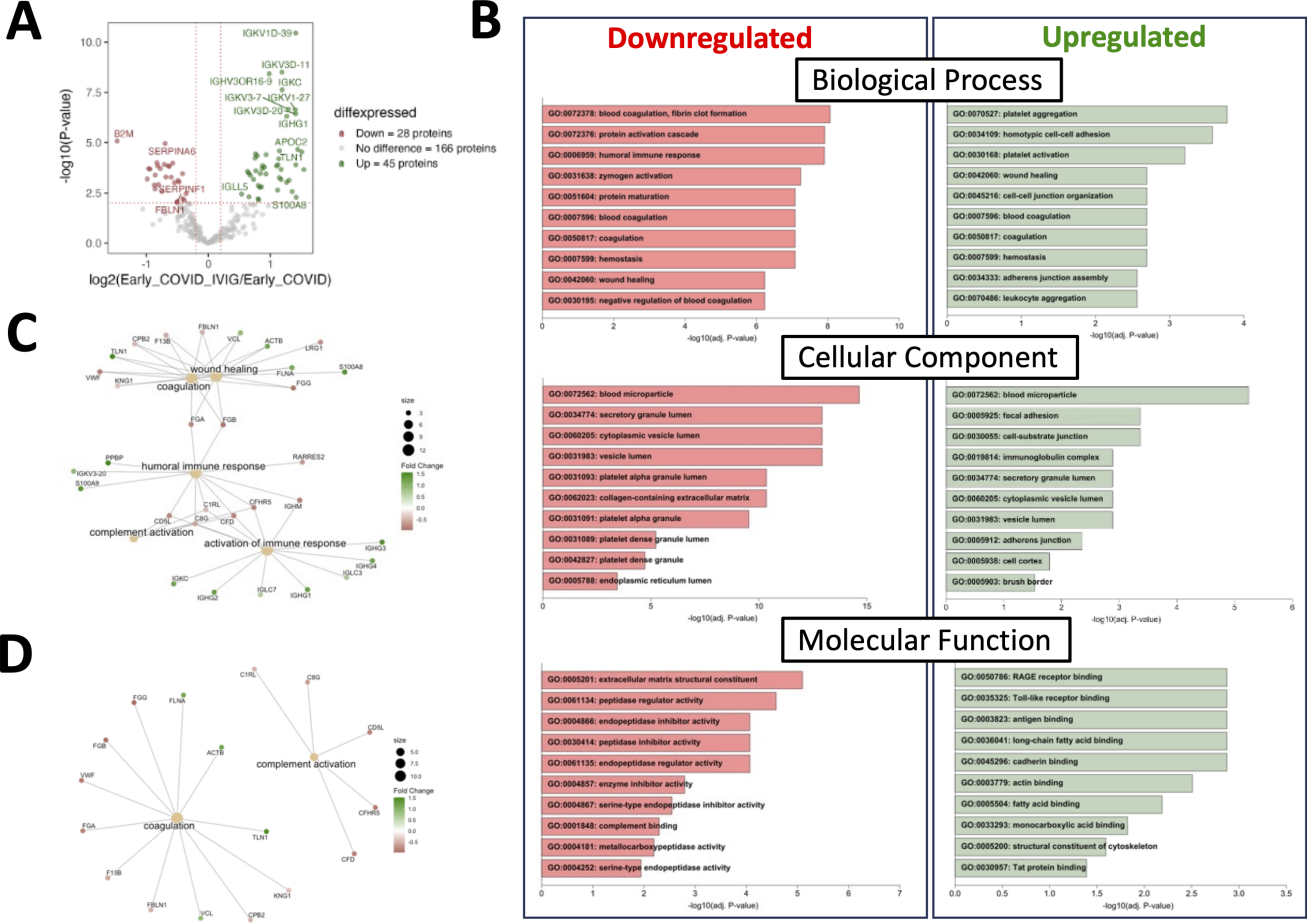
Treatment with IVIG is associated with numerous changes in coagulation-related pathways. Proteins from COVID-19 controls were compared to those from IVIG-treated patients at early time points. **(A)** A volcano plot representing proteins whose abundances significantly differed after IVIG treatment. **(B)** Gene ontology (GO) analysis showing downregulated and upregulated pathways. **(C)** Molecular network nodes highlighted according to drivers of GO terms, which include coagulation, complement activation, wound healing, activation of immune responses and humoral immune response pathways. **(D)** Molecular network nodes from coagulation and complement activation.

We then compared early samples from IVIG-treated COVID-19 patients to those from early COVID-19 controls. In this comparison, 28 proteins were downregulated and 45 upregulated in the IVIG group (**Figure 3A**). Notably, the differentially modulated proteins were predominantly associated with coagulation pathways, though changes were also observed in immune and complement-related proteins, including downregulation of complement-binding proteins and factors involved in wound healing (**Figure 3B**). This pattern was further supported by molecular network analysis (**Figure 3C**). Remarkably, pathway analysis revealed that several up- and downregulated pathways were the same, likely reflecting the significantly higher or lower abundance of specific proteins within these shared pathways in IVIG-treated patients compared to COVID-19 controls (**Figure 3B**). This becomes more apparent when examining a network pathway analysis focused specifically on the coagulation pathway (**Figure 3D**). In contrast, analysis of the same network for complement activation showed that most of the proteins with significant altered abundance are downregulated in IVIG-treated COVID-19 patients relative to COVID-19 controls (**Figure 3D**). Altogether, these findings suggests that IVIG modulates the coagulation cascade through both up and downregulation of several components, whereas its effect on the complement system appears to be more uniformly suppressive.

### Modulation of coagulation-specific proteins by IVIG treatment

**Figure 4:**
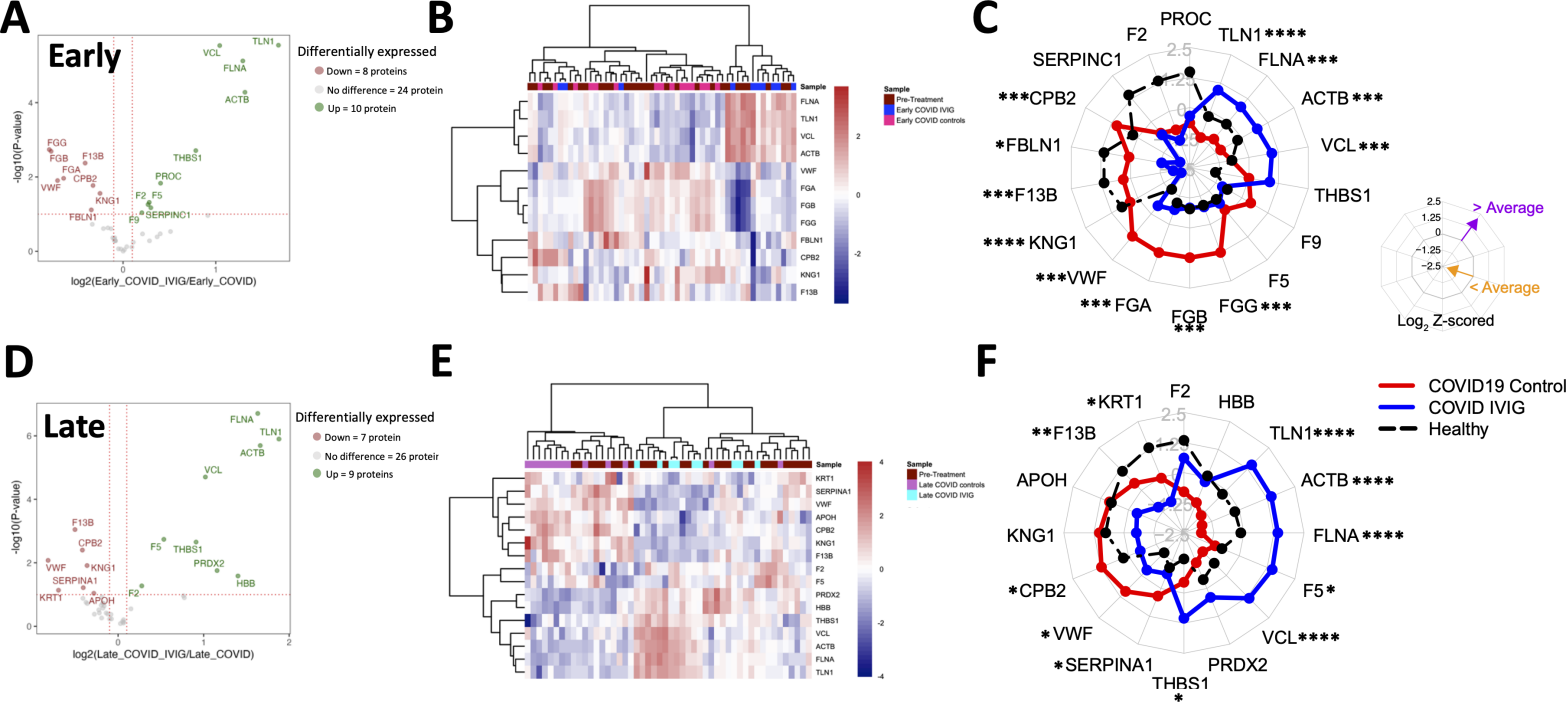
Overall modulation of coagulation-related proteins with IVIG treatment. Coagulation proteins differ between IVIG-treated patients compared to pretreatment, COVID-19 controls, and healthy controls, both at early and late time points. A volcano plot representing proteins whose abundances significantly differed in IVIG-treated patients when compared to COVID-19 controls at early **(A)** and late **(D)** time points. Heatmaps of proteins involving samples from COVID-19 controls and IVIG-treated subjects at early **(B)** and late **(E)** time points, as well as those from pre-treatment. Radar plots from early **(C)** and late **(F)** samples are presented as the subtraction to the mean log2 z-scores from each patient group (healthy controls [black], COVID-19 controls, and COVID-19 after IVIG [blue]). Vertices for radar plots were ordered based on hierarchical clustering to maximize differences between groups and significant differences are shown based on t-test analysis from the COVID-19 controls and COVID-19 IVIG-treated patients. * p < 0.05, ** p< 0.01, *** p < 0.001, **** p < 0.0001.

We decided to focus on both coagulation and complement pathways, as there is substantial evidence of the involvement of both in determining clinical outcomes in COVID-19 (35, 37, 42, 44, 50, 51). For both pathways, we analyzed the effects of IVIG treatment at early (**Figures 4A–C**) and later (**Figures 4D–F**) time points, which is relevant since IVIG was administered only over a 3–4 days course. At early time points following IVIG treatment, we found that patients upregulated 10 proteins and downregulated 8 proteins relative to COVID-19 controls (**Figure 4A**). A heatmap including the pre-treatment group showed some variability within each cohort, although early IVIG-treated patients tend to cluster with certain pre-treatment samples (**Figure 4B**). To better visualize the abundance differences in coagulation-related proteins, we constructed radar plots using healthy donors as a reference and recognized protein vertices based on hierarchical clustering. This approach more clearly highlighted group-level differences.

The radar plots showed that early after treatment, IVIG-treated patients exhibited increased levels of TNL1, FLNA, ACTB, and VCL, and decreased levels of FGG, FGB, FGA, VWF, F13B, FBLN1, CBP2, and KNG1 (**Figure 4C**). Interestingly, the relative abundance of FGG, FGB, FGA, F5, and F9 in IVIG-treated patients approximated levels found in healthy controls (**Figure 4C**). These trends were further illustrated in **Supplementary Figure 4A**, which compared relative abundance against pre-treatment values and reflected the same findings observed in the radar plots. **Table 2** details the differentially modulated coagulation-related proteins and their known pro- or anti-coagulant functions. Collectively, these results suggest that IVIG treatment modulates key coagulation-related proteins early in the disease course, in some cases restoring levels toward those observed in healthy controls.

When we analyzed samples collected at later time points (8–14 days post-treatment), we found 9 upregulated and 7 proteins downregulated in IVIG-treated patients relative to COVID-19 controls (**Figure 4D**). The heatmap analysis showed that coagulation-related protein abundances in IVIG-treated patients at this stage tended to cluster both with each other and with pre-treatment samples (**Figure 4E**). Similar to our observations at the early time point, the radar plot at later time points showed increased levels of TLN1, ACTB, FLNA, F5, VCL, and THBS1, and decreased levels of KRT1, F13B, CPB2, VWF, and SERPINA1 (**Figure 4F**, **Table 2**). When analyzing these profiles in the context of pre-treatment levels (supplemental **Figure 4B**), we found that IVIG-treated patients at later time points had relative abundances that remained closer to their pre-treatment state. Overall, these findings suggest that the impact of IVIG on coagulation-related proteins is most pronounced shortly after treatment, with a partial return to baseline levels observed at later time points. While significant modulation persists in some proteins, the overall magnitude of change diminishes over time.

### Modulation of complement-specific proteins by IVIG treatment

**Figure 5:**
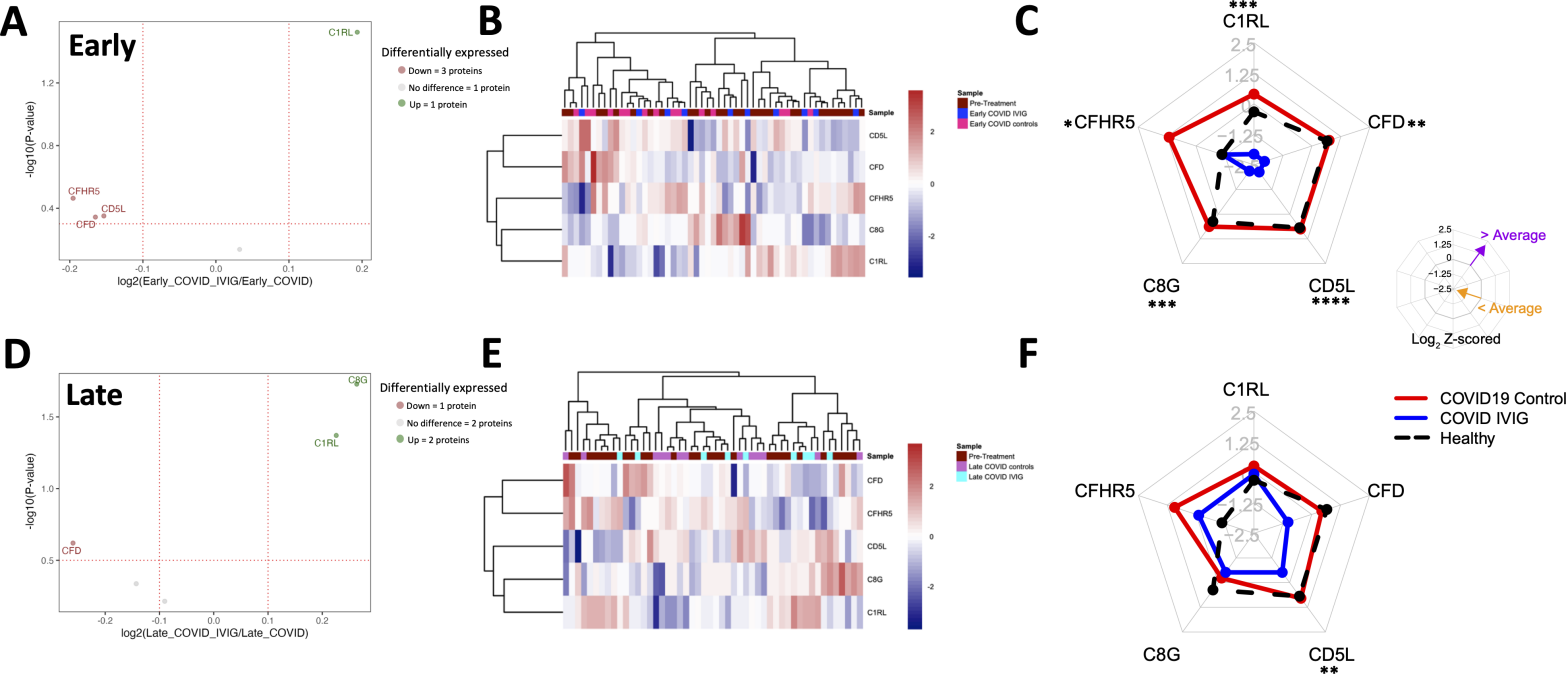
Overall modulation of complement-related proteins with IVIG treatment. Complement-related proteins were assessed based on the differences between IVIG-treated subjects compared to COVID-19 and healthy controls both at early and late time points. A volcano plot representing proteins whose abundances significantly differed from IVIG-treated patients to COVID-19 controls at early **(A)** and late **(D)** time points. Heatmaps of proteins from COVID-19 controls and IVIG-treated subjects at early **(B)** and late **(E)** time points, as well as those from pre-treatment. Radar plots from early **(C)** and late **(F)** samples are presented as the subtraction to the mean log_2_ z-scores from each patient group (healthy controls, COVID-19 controls and COVID-19 IVIG). Vertices for radar plots were ordered based on hierarchical clustering to maximize differences between groups and significant differences are shown based on t-test analysis from the COVID-19 controls and COVID-19 IVIG-treated patients. * p < 0.05, ** p< 0.01, *** p < 0.001, **** p < 0.0001.

For complement-related proteins, we found that IVIG-treated patients have the abundance of one protein increased and three proteins decreased compared to COVID-19 controls at early post-treatment time points (**Figure 5A**). Heatmap analysis showed high variability within each treatment group (**Figure 5B**). However, radar plots revealed a clear decrease in the average abundance of several complement proteins in IVIG-treated patients compared to both COVID-19 controls and healthy donors, including C1RL, CFD, CD5L, C8G, and CFHR5 (**Figure 5C**, **Table 2**). Similar findings were observed when comparing against pre-treatment samples: protein levels in IVIG-treated patients were lower than in both COVID-19 controls and pre-treatment samples (**Supplementary Figure 5A**).

At later time points following treatment, two complement proteins were increased and one was decreased in IVIG-treated patients (**Figure 5D**). Heatmap analysis again did not reveal a clear clustering pattern associated with treatment (**Figure 5E**). Unlike early time points, the radar plot shows similar average protein abundances across IVIG-treated subjects, COVID-19 controls, and healthy donors, with CD5L as the only protein showing a significant difference compared to COVID-19 controls (**Figure 5F**). This trend was also evident when comparing relative abundances, although the magnitude of differences was lower than at early time points (**Supplementary Figure 5B**). Interestingly, at early time-points, the complement protein profile of IVIG-treated patients resembles their pre-treatment baseline more closely than that of COVID-19 controls. These results suggest that IVIG modulates complement-related proteins transiently after treatment, with effects that are not sustained at later stages.

### IVIG treatment modulates key proteins from coagulation and complement pathways

**Figure 6:**
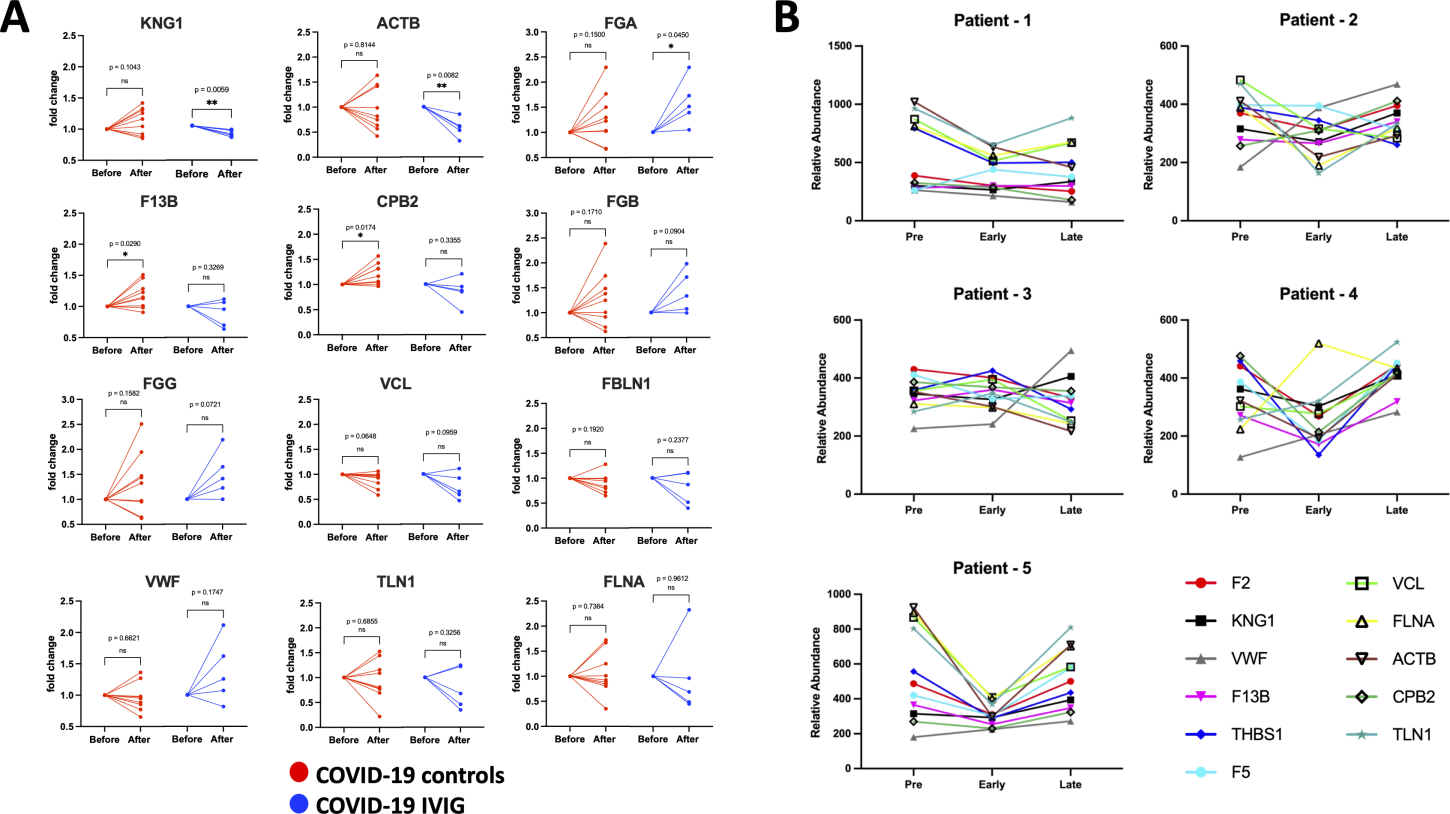
Individual modulation of coagulation-related proteins with IVIG treatment. Changes in relative protein abundances is shown as fold change from the pre-treatment time point (before) and early after treatment (after) for both COVID-19 controls (red; n=9) and COVID-19 treated with IVIG (blue; n=5). Proteins that showed change in abundance were evaluated using individual paired analyses. **(A)** Paired analysis for coagulation-related proteins. **(B)** Longitudinal analysis of relative abundances of each protein from pre-treatment, as well as early and late time points after treatment. P-values from paired analysis were estimated using paired t-test (before versus after) for each group. * p < 0.05, ** p < 0.01 ns is for no significant.

Since the most pronounced changes following IVIG treatment occurred early, likely due to the short 3–4 days treatment course—we focused on paired analyses comparing pre-treatment and early post-treatment samples. In the coagulation pathway, we found that IVIG treatment decreases KNG1 and ACTB and increased FGA relative to COVID-19 controls (**Figure 6A**), although the variance in FGA levels was similar between groups. Additionally, F13B and CPB2 increased in COVID-19 controls but not in IVIG-treated patients. No significant changes were observed for FGB, FGG, VCL, FBLN1, VWF, TLN1, and FLNA at the individual level (**Figure 6A**), though some of these proteins were significantly reduced in unpaired group-level analyses (**Figure 5C**, **Supplementary Figure 4A**).

Longitudinal analysis of relative protein abundance across pre-treatment, early, and late time points showed that reductions in coagulation-associated proteins were most evident early after IVIG treatment and largely returned to baseline by the later time point (**Figure 6B**). However, the magnitude and direction of the response varied between individuals. These data suggest that IVIG transiently reduces proteins known to promote coagulation (**Table 2**), though this effect may not persist into later stages of illness.

**Figure 7:**
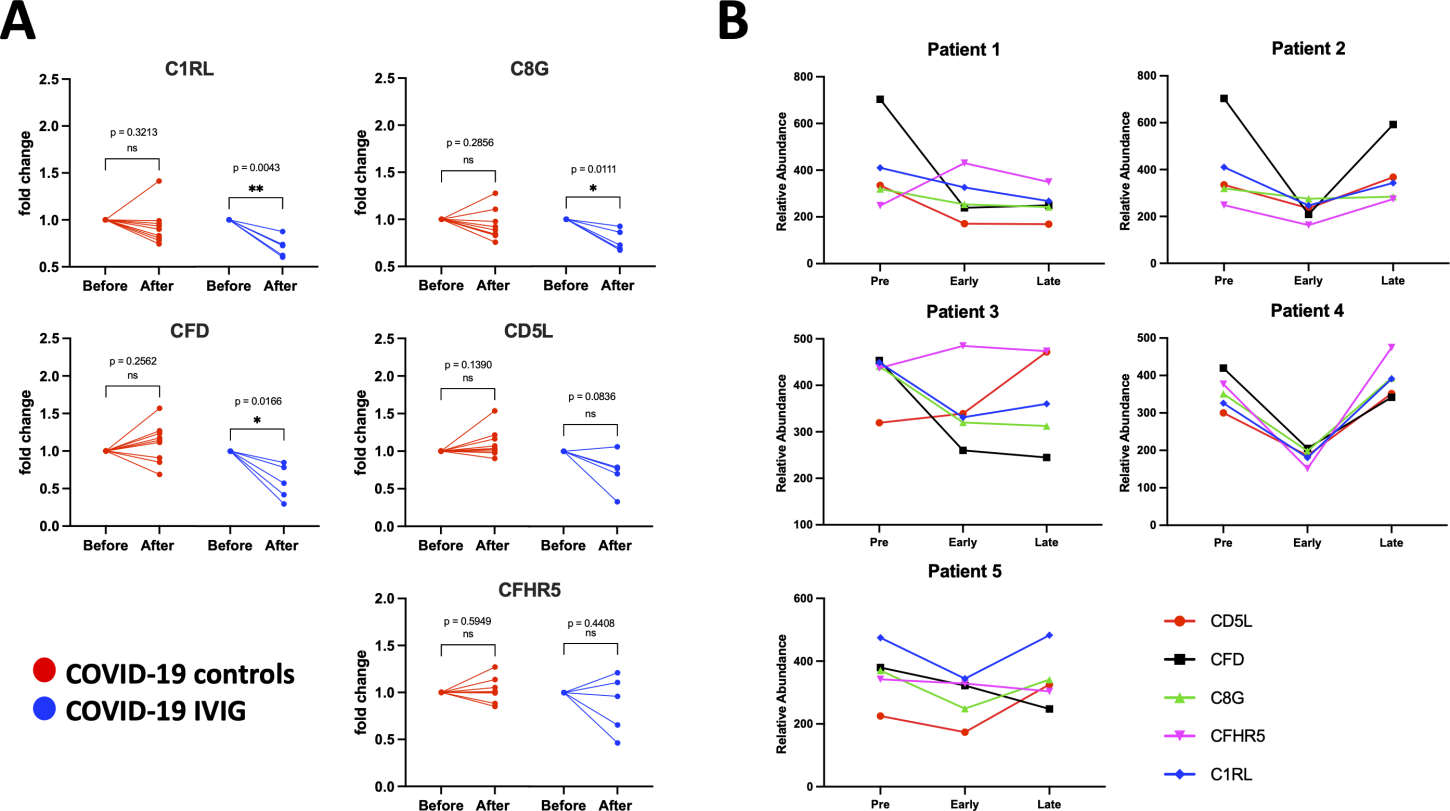
Individual modulation of complement-related proteins with IVIG treatment. Changes in relative protein abundances are shown as fold change from pre-treatment (before) and early after treatment (after) for both COVID-19 controls (red; n=9) and COVID-19 IVIG (blue; n=5). Proteins that showed change in abundance were evaluated using individual paired analyses. **(A)** Paired analysis for complement-related proteins. **(B)** Longitudinal analysis from relative abundances of each protein from pre-treatment to early and late time points upon treatment. P-values from paired analysis were estimated using paired t-test (before versus after) for each group. * p < 0.05, ** p < 0.01 ns is for no significant.

In the complement pathway, IVIG treatment decreased levels of C1RL, C8G, and CFD compared to COVID-19 controls (**Figure 7A**). These proteins are known to promote complement cascade activation (**Table 2**). In contrast, CD5L and CFHR5 did not differ significantly between groups (**Figure 7A**). As with the coagulation proteins, longitudinal analysis showed that complement-related proteins were reduced shortly after IVIG treatment, but these effects were not sustained at later time points (**Figure 7B**). The pattern varied among patients, again suggesting a transient and heterogeneous effect of IVIG on complement activation.

## Discussion

COVID-19 ARDS is marked by exaggerated immune responses and coagulopathic complications (35, 52). Early in the pandemic, various immunomodulatory therapies—including steroids, targeted biologics, and antibody-based treatments such as convalescent plasma, interleukin antagonists, and IVIG—were deployed empirically (53, 54). Given its established efficacy in autoimmune and inflammatory diseases, IVIG emerged as a candidate therapy, particularly prior to the availability of COVID-19 vaccines. Its standard-of-care use in Kawasaki disease and multisystem inflammatory syndrome associated with COVID-19 further supported this rationale. All patients in our cohort were unvaccinated, thus eliminating a confounding effect from prior immunological memory.

Consistent with prior studies (13, 14, 28, 30–34), we found that IVIG did improve overall survival compared to COVID-19 controls and was associated with longer hospitalizations an observation also noted in other cohorts (14, 32). However, aggregate outcome data may observe clinically important effects. Specifically, our results demonstrate that early IVIG administration was associated with improved outcomes: increased survival rates, better oxygenation, and greater functional recovery at discharge and (18, 21, 24, 55). Patients who received IVIG earlier were more likely to be discharged home rather than to a convalescent facility, and delayed administration correlated with worse functional status and higher mortality in a time-dependent manner. These findings suggest that the timing of IVIG therapy is a critical determinant of clinical benefit, and may explain why prior trials that did not account for treatment timing failed to capture efficacy.

The inconsistent outcomes observed across studies evaluating IVIG in severe COVID-19 disease likely reflect variation in multiple factors: dosing regimen, formulation, timing, and patient heterogeneity, including pre-existing co-morbidities. IVIG dosing in published studies has ranged from 0.1 to 1 g/kg over durations of 2 to 9.5 days, with treatment initiated anywhere between day 2 to 22 of hospitalization (56). Efficacy may also depend on IVIG characteristics, including concentration, stabilizers, glycosylation profiles, plasma source, and the presence of specific immunoglobulin fractions (57, 58). Differences in formulation (*e.g.* IgG- *vs*. IgM-enriched) may further influence outcomes. In our study, IVIG was administered in a relatively standardized fashion (0.5 g/kg/day for 4 days), and the proteomic analysis provides insight into its potential mechanisms of action.

The exact mechanism of action of IVIG is difficult to define due to the wide variety of interactions that antibodies can have. This complexity can be explained by the network theory of the immune system, which suggests that immune cells and molecules respond to and regulate each other through specific chemical interactions within a network (59, 60). Based on this theory, it has been proposed that IVIG mitigates severe COVID-19 by containing suppressive antibodies within a symmetrically balanced network structure that interacts with the imbalanced immune network of the infected patient (61). It has also been proposed that anti-idiotypic antibodies may serve as powerful therapeutic agents by neutralizing specific pathogenic antibodies (62–64). Thus, the timing at which IVIG is given to potentially neutralize detrimental autoantibodies could be essential to ameliorate collateral immunopathology observed in COVID-19.

To investigate other potential mechanisms by which IVIG provides protection in COVID-19—beyond the effects of anti-idiotypic antibodies—we conducted the first proteomic analysis of plasma from IVIG-treated COVID-19 patients, in which immunoglobulins had been depleted. Prior plasma proteomic studies in COVID-19 have identified signatures associated with inflammation, coagulation, complement activation, platelet degranulation, and lipid metabolism, all of which likely contribute to disease pathogenesis (65, 66). Our findings replicated these findings in COVID-19 controls; and revealed that IVIG treatment, particularly when administered early, partially reversed or attenuated dysregulation in coagulation and complement pathways (65). In fact, coagulation-related proteins, represented the majority of significantly modulated targets following IVIG, suggesting that modulation of the coagulation cascade may underly observed clinical improvements, especially with early intervention.

Many of the coagulation-related proteins we identified have been previously shown to be elevated in COVID-19 and enriched in extracellular vesicles (67). Although IVIG also reduced complement-associated proteins, the magnitude of this effect was smaller than those for coagulation targets. Nevertheless, given the well-established crosstalk between the complement and coagulation systems (68), even modest effects on complement may contribute to clinical benefit. Dysregulation in these overlapping pathways likely contributes to the hypercoagulable state injury to the microvasculature of critical organs in severe COVID-19 (69).

IVIG therapy has previously been shown to affect the coagulation system. In sepsis, IVIG was associated with reduced inflammatory responses and improved coagulation markers; although a trend toward reduced mortality did not reach statistical significance in one small study (70), a study with a larger cohort reported significantly improved 28-day survival in patients with sepsis-induced coagulopathy (71). This supports the concept of IVIG as an adjunct therapy for sepsis-induced coagulopathy. In contrast to conventional sepsis, therapeutic anticoagulation improves survival in COVID-19, whereas prophylactic dosing is often inadequate (72). IVIG may reduce thrombus formation by attenuating the hypercoagulable state. Coagulopathy increases mortality in COVID-19, with pulmonary thrombosis being the most frequent event, though deep venous thrombosis, *in situ* microvascular thrombosis, and macrovascular thrombosis are also observed (73). Interestingly, in Kawasaki disease—a condition treated with IVIG—coagulation abnormalities are common but mitigated by early IVIG therapy (45). However, patients in a hypercoagulable state may be less responsive to IVIG, potentially due to consumption resistance mechanisms (45, 74). This may explain the lack of benefit seen in late-stage disease. It is important to note that several IVIG drug insert packages warn of a risk of thromboembolism (75), particularly in patients with paralysis, obesity, immobility, prior thromboembolic events, hypertension, diabetes, or advanced age (76–78). Some of these comorbidities were present in our subjects and associated with severe COVID-19 disease. Older IVIG formulations were more thrombogenic due to higher concentrations of clotting factors (II, VII, IX, X, and particularly XIa), although modern manufacturing has reduced this risk (79–81).

In our proteomic data, IVIG treatment led to early reductions in multiple procoagulant proteins, including KNG1, ATCB, FGA, F13B, and CPB2. KNG1, a precursor to bradykinin and a component of the intrinsic coagulation cascade, is elevated in COVID-19 and contributes to both inflammation and thrombosis (82–84). ACTB (β-actin), normally intracellular, can appear extracellularly during neutrophil extracellular trap formation, cell injury, or release of extracellular vesicles and exosomes, and is associated with endothelial damage and hypercoagulability (85–87). FGA, the α-chain of fibrinogen, is a thrombus precursor and elevated in severe COVID-19 (88); its early increase in IVIG-treated patients may reflect fibrin turnover. F13B stabilizes the active transglutaminase F13A in plasma but may accumulate independently in COVID-19, where its function is less clear (67, 89–91). CBP2, a regulator of fibrinolysis, may impair clot breakdown during COVID-19 infection and elevate thrombosis risk (92). We observed reduced CPB2 levels in IVIG-treated patients relative to COVID-19 controls. Together, these findings suggest that IVIG may transiently normalize hypercoagulation signatures in early disease.

Considering the effect of IVIG on complement, prior studies have found that it contains antibodies with complement scavenging activity, providing beneficial effects in autoimmune diseases such as dermatomyositis and Kawasaki disease (38–41). IVIG has also been shown to inhibit complement activation in a dose-dependent manner shortly after infusion, however, this effect appears short-lived, with complement activity returning to baseline within 2–4 weeks (93). In our study, we similarly found that changes in both coagulation- and complement-related proteins were most apparent at early time points after treatment, and were attenuated or resolved by later stages, returning toward pre-treatment levels.

Although the five complement-associated proteins reduced after IVIG treatment—CD5L, CFD, C8G, CFHR5, and C1RL—only C1RL, C8G, and CFD showed statistically significantly decreased in paired pre- and post-treatment analyses. C1RL is a component of the classical complement pathway (94), although its specific role in COVID-19 remains poorly characterized. C8G is a subunit of complement component C8, part of the membrane attack complex (MAC) (95)—a cytolytic structure composed of C5b, C6, C6, C8, and C9—that has been implicated in severe COVID-19 (96, 97). CFD is essential in both the initiation and amplification phases of the alternative complement pathway (98) and is increased in severe COVID-19 cases (99). Notably, direct activation of the alternative pathway by the SARS-CoV-2 spike protein can be blocked by CFD inhibition (100).

These findings support a model in which dysregulation of both the classical and alternative complement pathways contribute to poor outcomes in COVID-19 by promoting systemic inflammation and thrombosis (98). IVIG appears to modulate several key components of complement activation, potentially mitigating this dysregulation in a subset of patients. In support of the mechanism, targeted inhibition of C5a with vilobelimab has shown survival benefit in a randomized trial of critically-ill COVID-19 patients when administered within 48 hours of mechanical ventilation (101), underscoring the role of complement in disease pathogenesis.

This study represents the first proteomic analysis of plasma from IVIG-treated COVID-19 patients, and the first to demonstrate that simultaneous modulation of coagulation and complement proteins may underlie the benefit of early IVIG therapy in severe COVID-19. However, several limitations must be acknowledged. The cohort size was small, and the number of patients with paired samples at pre-, early, and late time points was even smaller, limiting statistical power. Additionally, samples were collected from residual clinical blood draws rather than uniformly timed research protocols, preventing correlation analysis between specific protein changes and clinical outcomes.

Despite these limitations, our findings are supported by mechanistic parallels in non-COVID-19 disease models. These data suggest that therapies targeting immunothrombosis and immune-associated tissue damage may be useful in treating ARDS and vascular complications in severe COVID-19. Moreover, the impact of IVIG on shared pathogenic pathways suggests its potential relevance for ARDS caused by other viral infections. As with many acute interventions, early administration appears to be critical to achieving benefit. Future studies should evaluate host-directed factors to guide allocation of IVIG in a cost-effective and mechanistically informed manner.

## Data Availability

The datasets presented in this study can be found online at the Zenodo.org: https://doi.org/10.5281/zenodo.15883238.

## Supplementary material

Supplementary Figure 1 COVID-19 controls and COVID-19 IVIG have similar APACHE II score at admission. APACHE II scores values from COVID-19 controls and COVID-19 IVIG, compared via t-test.

Supplementary Figure 2 Modulation of proteome over time in IVIG-treated patients. PCA performed on samples from IVIG treated subjects at early and late time points during treatment as well as pre-treatment, compared to healthy controls.

Supplementary Figure 3 Severe COVID-19 disease increases inflammatory and coagulation proteins in the circulation. Proteins from COVID-19 controls (at pre-treatment phase) were compared to those from healthy controls. **(A)** A volcano plot representing proteins whose abundances significantly differed from healthy controls. **(B)** Gene ontology (GO) analysis showing downregulated and upregulated pathways. **(C)** Molecular network nodes highlighted according to drivers of GO terms, which include activation of immune response, acute inflammatory response, complement activation and coagulation pathways.

Supplementary Figure 4 Comparison of changes to specific coagulation-related proteins at early and late time points. Differences in relative abundance between coagulation-related proteins in COVID-19 controls and COVID-19 IVIG in **(A)** early and **(B)** late time points, as well as pre-treatment levels. The same pre-treatment values are shown in both “early” and “late” protein levels as baseline prior IVIG treatment. P-values were defined via one-way ANOVA with Tukey’s multiple comparisons test.

Supplementary Figure 5 Comparison of changes to specific complement-related proteins at early and late time points. Differences in relative abundance between complement-related proteins COVID-19 controls and COVID-19 IVIG at **(A)** early and **(B)** late time points as well as pre-treatment values. The same pre-treatment values are shown in both “early” and “late” protein levels as baseline prior IVIG treatment. P-values were defined via one-way ANOVA with Tukey’s multiple comparisons test.
