# Supplementary figures and images for: Treatment with intravenous immunoglobulin modulates coagulation- and complement-related pathways in COVID-19 patients
Source: Front Immunol. 2025 Jul 31;16:1623309. doi: 10.3389/fimmu.2025.1623309 (PMC12350128; doi:10.3389/fimmu.2025.1623309)

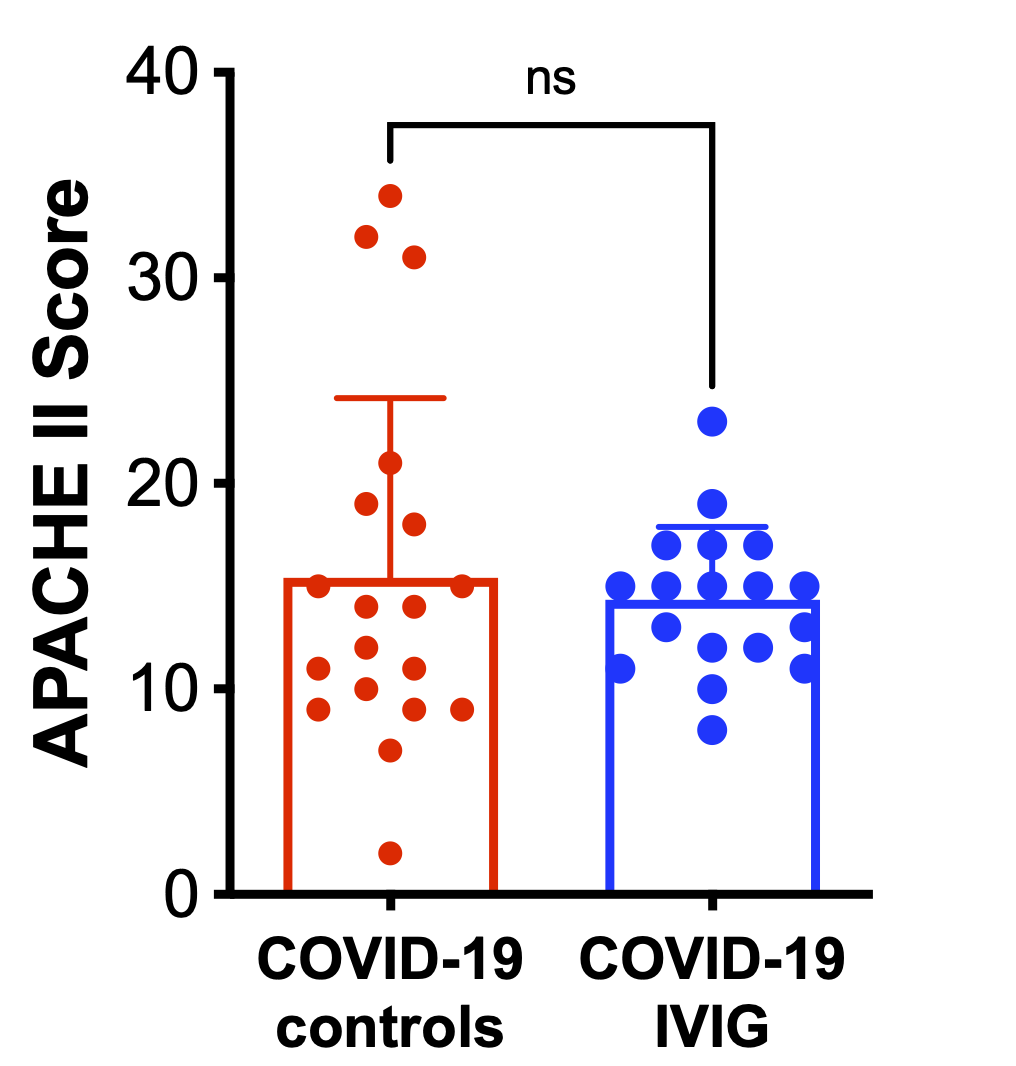

Supplement: Supplementary Figure 1 — COVID-19 controls and COVID-19 IVIG have similar APACHE II score at admission. APACHE II scores values from COVID-19 controls and COVID-19 IVIG, compared via t-test. [file Image1.tiff]

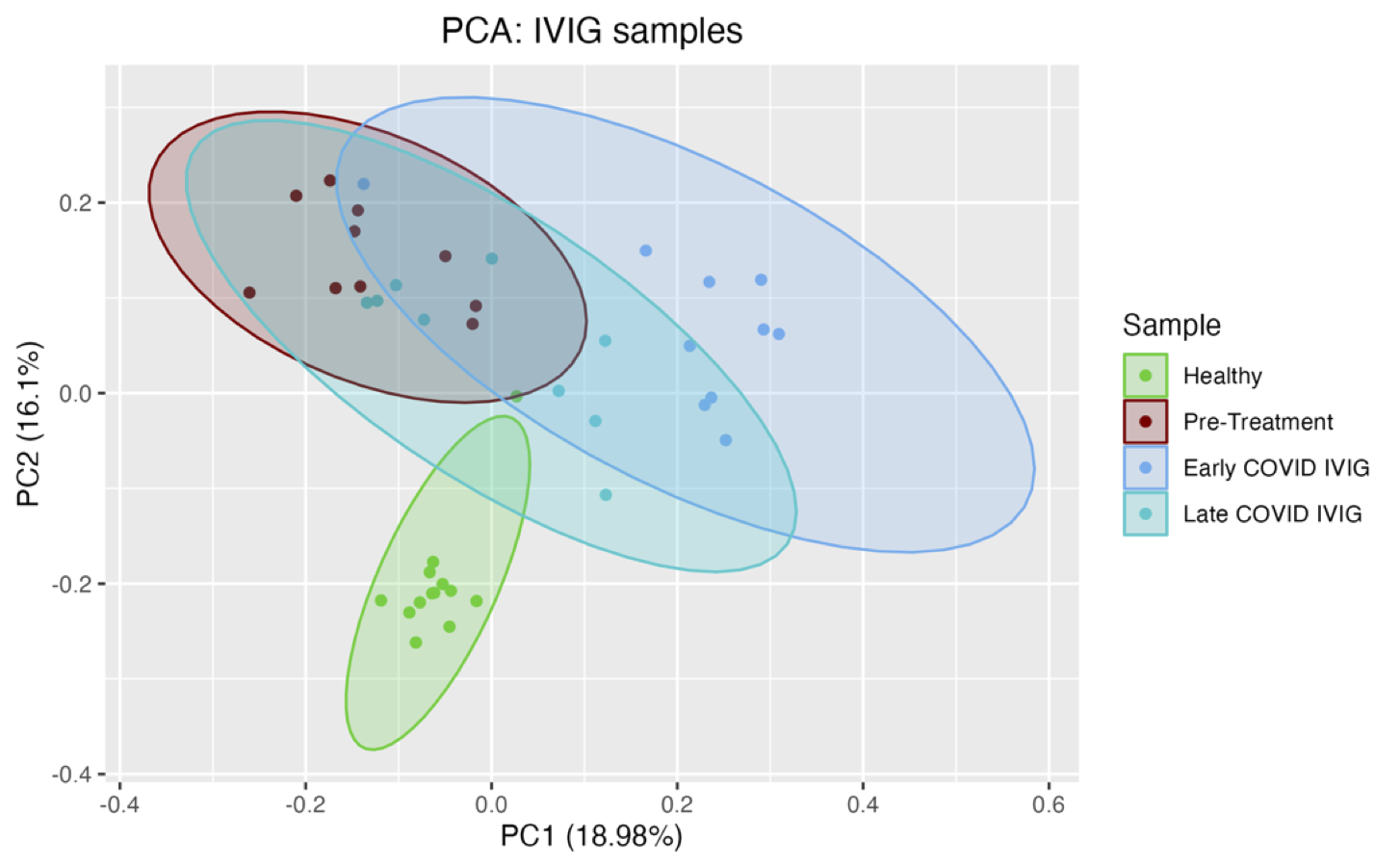

Supplement: Supplementary Figure 2 — Modulation of proteome over time in IVIG-treated patients. PCA performed on samples from IVIG treated subjects at early and late time points during treatment as well as pre-treatment, compared to healthy controls. [file Image2.tiff]

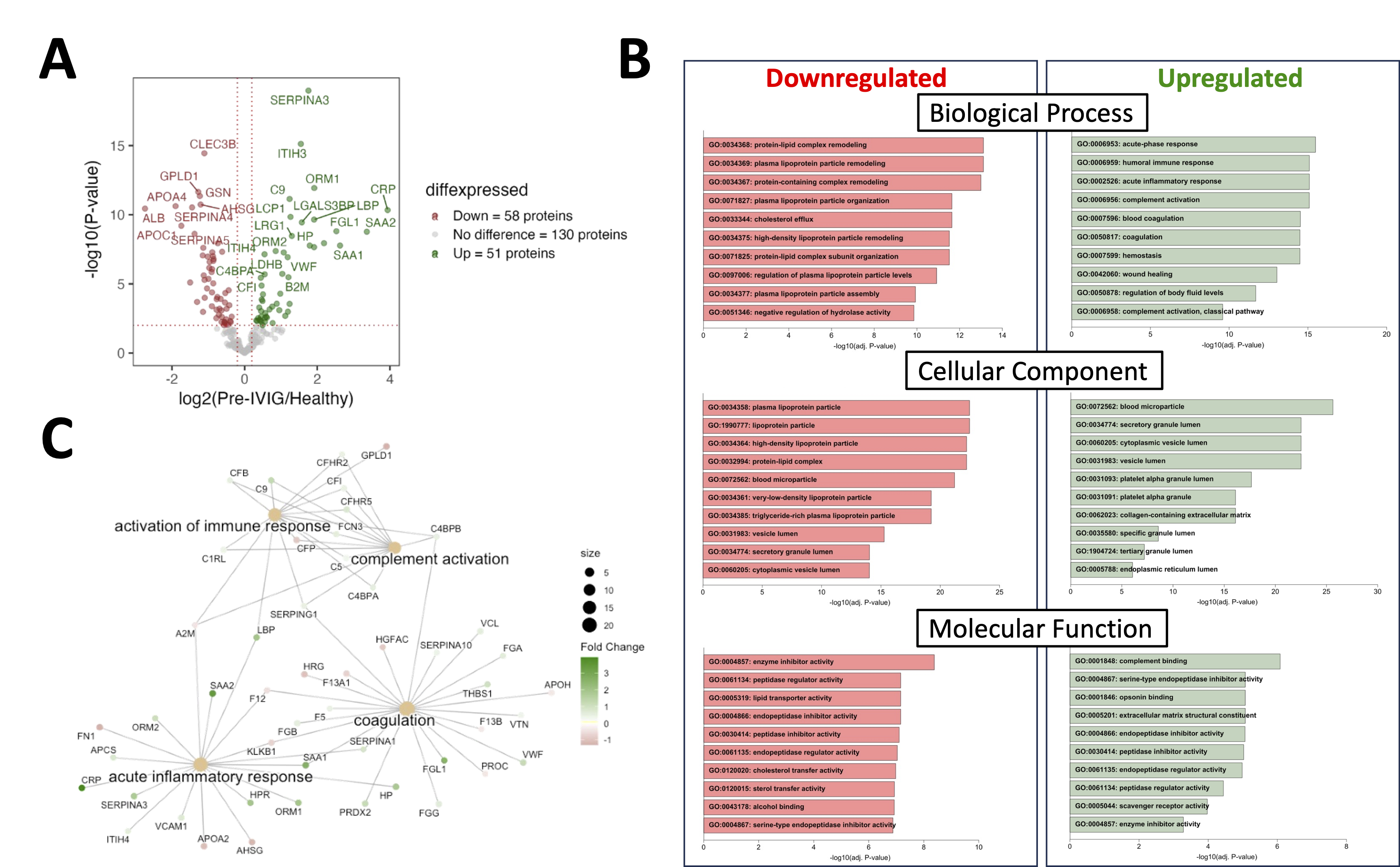

Supplement: Supplementary Figure 3 — Severe COVID-19 disease increases inflammatory and coagulation proteins in the circulation. Proteins from COVID-19 controls (at pre-treatment phase) were compared to those from healthy controls. (A) A volcano plot representing proteins whose abundances significantly differed from healthy controls. (B) Gene ontology (GO) analysis showing downregulated and upregulated pathways. (C) Molecular network nodes highlighted according to drivers of GO terms, which include activation of immune response, acute inflammatory response, complement activation and coagulation pathways. [file Image3.tiff]

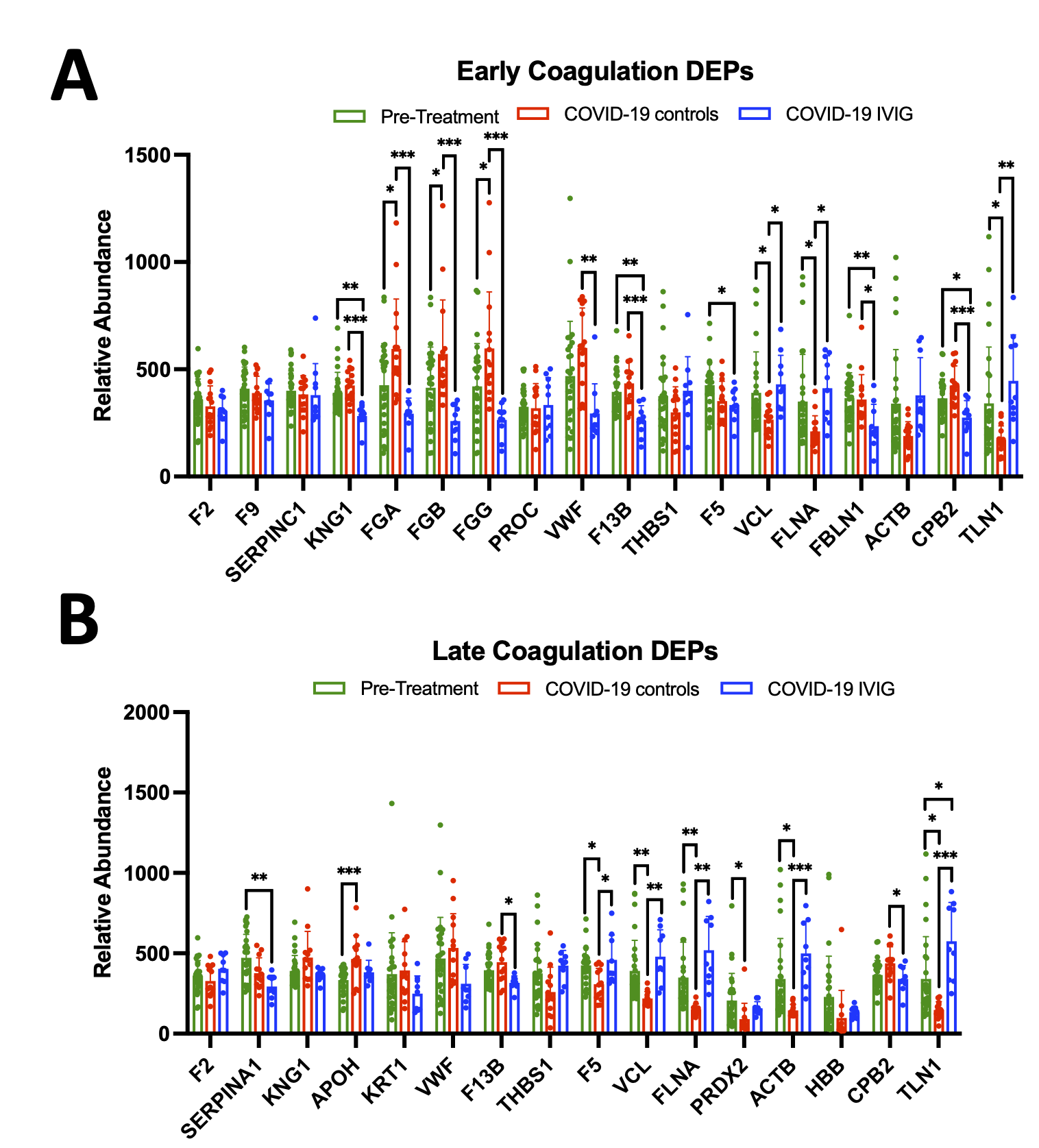

Supplement: Supplementary Figure 4 — Comparison of changes to specific coagulation-related proteins at early and late time points. Differences in relative abundance between coagulation-related proteins in COVID-19 controls and COVID-19 IVIG in (A) early and (B) late time points, as well as pre-treatment levels. The same pre-treatment values are shown in both “early” and “late” protein levels as baseline prior IVIG treatment. P-values were defined via one-way ANOVA with Tukey’s multiple comparisons test. [file Image4.tiff]

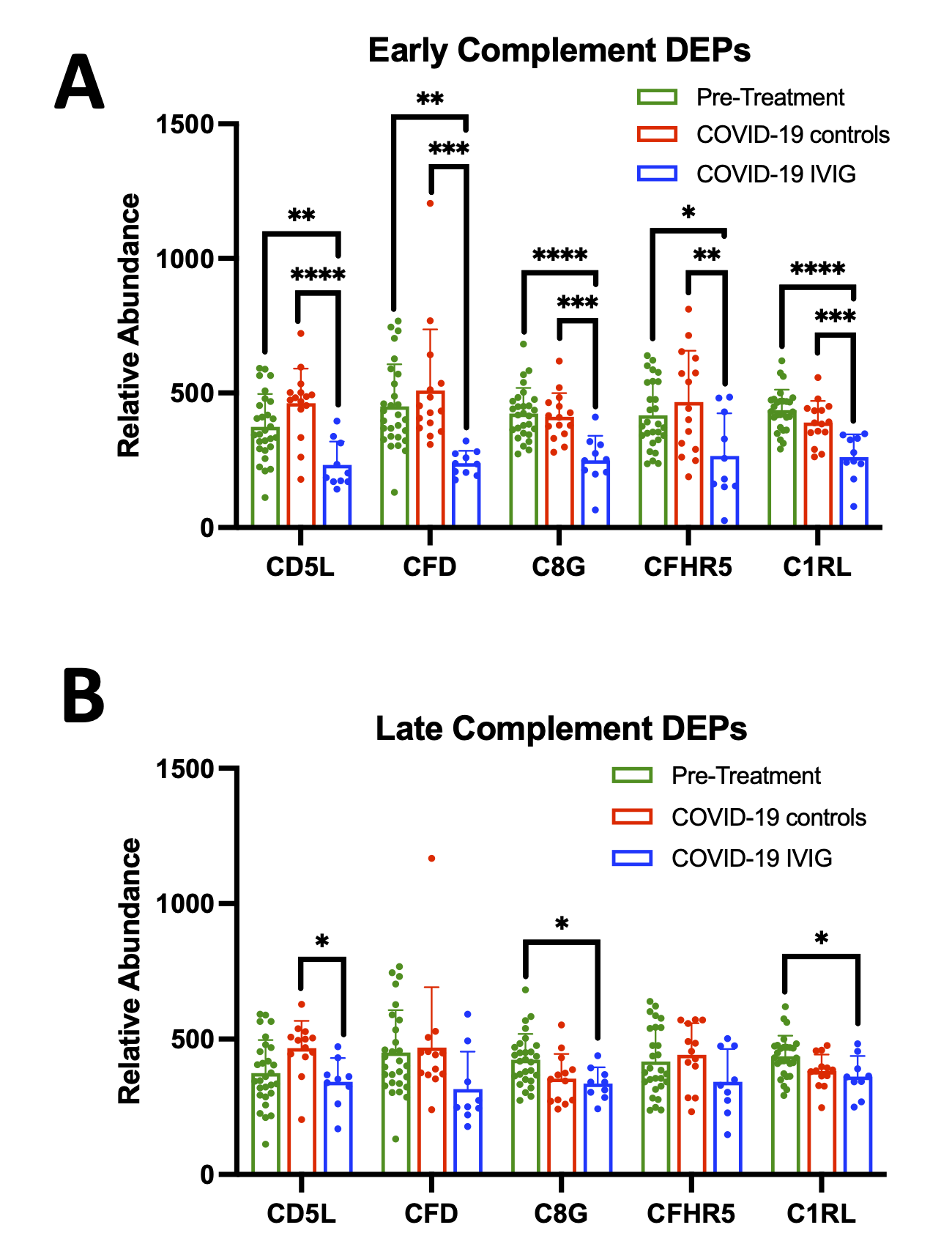

Supplement: Supplementary Figure 5 — Comparison of changes to specific complement-related proteins at early and late time points. Differences in relative abundance between complement-related proteins COVID-19 controls and COVID-19 IVIG at (A) early and (B) late time points as well as pre-treatment values. The same pre-treatment values are shown in both “early” and “late” protein levels as baseline prior IVIG treatment. P-values were defined via one-way ANOVA with Tukey’s multiple comparisons test. [file Image5.tiff]
